# Measurement invariance of the 10-item resilience scale specific to cancer in Americans and Chinese: A propensity score–based multidimensional item response theory analysis

**DOI:** 10.1016/j.apjon.2022.100171

**Published:** 2022-11-26

**Authors:** Muzi Liang, Peng Chen, Alex Molassiotis, Sangchoon Jeon, Ying Tang, Guangyun Hu, Yunfei Zhu, Zhe Sun, Yuanling Yu, Tish M. Knobf, Zengjie Ye

**Affiliations:** a Guangdong Academy of Population Development, Guangzhou, China; b Basic Medical School, Guizhou University of Traditional Chinese Medicine, Guiyang, China; c College of Arts, Humanities & Education, University of Derby, Derby, UK; d School of Nursing, Yale University, Orange, CT, USA; e Institute of Tumor, Guangzhou University of Chinese Medicine, Guangzhou, China; f Army Medical University, Chongqing, China; g Shenzhen People's Hospital, Shenzhen, China; h The First Affiliated Hospital, Guangzhou University of Chinese Medicine, Guangzhou, China; i South China University of Technology, Guangzhou, China; j Guangzhou University of Chinese Medicine, Guangzhou, China

**Keywords:** Measurement invariance, Resilience, RS-SC-10, Americans, Chinese, Propensity score matching, Multidimensional item response theory

## Abstract

**Objective:**

Little is known about the measurement invariance (MI) of resilience instruments in cancer care. This study was designed to examine MI of 10-Item Resilience Scale (RS-SC-10) in Americans and Chinese with cancer using propensity score–based multidimensional item response theory (MIRT) analysis.

**Methods:**

A sample of 924 patients were enrolled in the Be Resilient to Cancer trial involving 1 hospital in America and 3 hospitals in China. Data were collected from the RS-SC-10 and Hospital Anxiety and Depression Scale. Propensity score matching and MIRT were performed to evaluate Differential Item Function. Integrated Discrimination Improvement and Net Reclassification Improvement were used to indirectly estimate the MI through incremental prediction ability of MIRT-based score over total score.

**Results:**

RS-SC-10 retained 10 items with monotonous thresholds and its original two-factor structure. Nonuniform Differential Item Function was recognized in Item 4 (*P* ​= ​0.0011, Δ%β1 ​= ​4.15%) and Item 8 (*P* ​= ​0.0017, Δ%β1 ​= ​5.99%). Net Reclassification Improvement ranged from 9.04% to 35.01%, and Integrated Discrimination Improvement ranged from 8.82% to 20.60%.

**Conclusions:**

Although partial MI has been identified between Americans and Chinese, RS-SC-10 remains a critical indicator to emotional distress in cancer care.

## Introduction

Despite significant improvements in 5-year survival,^1^^,^^2^ psychosocial distress (eg, depression, anxiety, fear of cancer recurrence) persists in cancer survivors, which is associated with a poorer quality of life (QoL).^3^^,^^4^ Resilience, defined as the ability to bounce back after a traumatic event, has become an important concept in research across disciplines.^5^ For example, cancer survivors with higher resilience levels were reported to have lower psychological distress as well as better QoL.^6^ Thus, resilience may be a critical indicator of QoL and data support use in descriptive and intervention studies. A new resilience instrument, the Resilience Scale Specific to Cancer (RS-SC), was developed based on Shift-Persist theory and Resilience Model to Breast Cancer.^7^^,^^8^ Subsequent to the original 25-item scale, a shortened version was created, the 10-item RS-SC (RS-SC-10) based on Item Response Theory (IRT) analysis,^9^^,^^10^ and validated in our Be Resilient to Breast Cancer trial.^11^^,^^12^ RS-SC-10 is a psychometrically established resilience instrument for cancer survivor. We wanted to determine if RS-SC-10 had the property of measurement invariance (MI) in different ethnic groups (ie, Americans vs. Chinese), which is defined as the instrument capturing the same underlying construct across distinct groups or period.^13^ Patients can vary in their interpretations, understanding, and conceptualization of some items because of the differences in language or cultural assumptions.^14^ For example, because of the difference in cultural background, Americans and Chinese may read, interpret, and answer “item 9: *I believe that good fortune will come after surviving a disaster, derived from RS-SC-10*” quite differently. If we compare Americans and Chinese on the same scale, the “actual difference” in scoring may not be attributed to a “true difference.” These differences can be sources of bias resulting in nonequivalent constructs across ethnically diverse groups. In addition, confounders such as unbalanced demographics in most MI-based research are often neglected, resulting in biased parameter estimation in MI.^15^^,^^16^ In the present study, Propensity score matching (PSM) was performed for a confounder-balanced comparison and then followed by a multidimensional item response theory (MIRT)-based Differential Item Function (DIF) analysis. In addition, exploratory indicators, including Net Reclassification Improvement (NRI) and Integrated Discrimination Improvement (IDI), were used in the Resilience-Distressed prediction models (MIRT-based score vs. total score) to provide additional information about MI between Americans and Chinese. MI could be confirmed if similar NRI and IDI were identified between Americans and Chinese, indicating that MIRT-based score had higher net benefits and better predictable abilities to the distressed outcome than the resilience total score. We hypothesized that (1) RS-SC-10 would retain its original two-factor structure in Americans and Chinese; (2) uniform/nonuniform DIF would be identified in some items between Americans and Chinese; (3) similar NRI and IDI would be identified between Americans and Chinese and MIRT-based score could offer incremental predictive value above total score.

## Methods

### Language equivalence

The cross-cultural translation of RS-SC-10 was performed according to World Health Organization's guideline. Two independent Chinese-English native-speaking researchers translated the Chinese version into English.^17^ The two translated scales were compared, and a consensus was reached by a panel of experts in the field of cancer care. The consensus version was then back-translated by a third bilingual English-Chinese researcher, and the back translation English version was reviewed against the original Chinese version. No significant discrepancies were identified, and the final version was approved and sent to 15 Americans with cancer for final validation.

### Patients and data collection

One hospital in America and 3 hospitals in China participated in the present study. A potential sample of 1052 patients were initially approached between March 2018 and May 2021. Patients with missing demographic or instrument data were excluded, resulting in a final sample of 924 (87.8%). The inclusion criteria were (1) confirmed diagnosis of cancer, (2) aged > 18 years, and (3) receiving active treatment. The exclusion criteria were (1) linguistic or intellectual difficulties, (2) had a currently active Axis I psychiatric disorder, and (3) unwilling to participate in the study. The patients were approached by trained research nurses, and instruments were administered following informed consent.

### Ethical approval

The present study was part of the Be Resilient to Cancer, and the details of ethic approval have been described.^18^, ^19^, ^20^

### Instruments

#### Ten-item resilience scale specific to cancer

RS-SC-10 is derived from the original 25-item RS-SC, which has five domains of Generic Element, Benefit Finding, Support and Coping, Hope for The Future, and Meaning for Existence.^7^ RS-SC-10 has two domains: Generic Elements and Shift-Persist.^10^^,^^21^ It is a 5-point Likert scale, with higher scores indicating higher resilience levels (total score ranges from 10 to 50). RS-SC-10 was attached in the appendix file.

#### Hospital anxiety and depression scale

Hospital Anxiety and Depression Scale (HADS) contains 7 items for anxiety and 7 items for depression, respectively, with higher scores indicating higher levels of anxiety and depression.^22^ In this present study, a cutoff of 7 was applied to select patients with high anxiety or depression.^23^ Patients indicated by anxiety or depression ≥ 7 were defined as distressed (outcome ​= ​1), whereas others were defined as nondistressed (outcome ​= ​0).

#### Data analysis

First, the propensity score was calculated by multivariable logistic regressions, and demographics, including gender, education, cancer type, stage of cancer, and time since diagnosis associated with resilience, were matched in PSM. To maximize the ratio while maintaining a 90% match rate in the Be Resilient to Cancer group, we used a 1-to-2 (Americans to Chinese) matching design using the nearest neighbor method within a caliper of 0.1.^24^ Absolute standardized difference < 0.1 indicates a good balance. PSM methods were successfully performed in our previous research.^19^^,^^25^ Second, local independence was examined by item-pair residual correlations, and less than 0.2 is recommended.^26^ Third, Confirmatory Factor Analysis–based and Bifactor-based MIRT models were explored in the present study.^27^ A compensatory logistic multidimensional grade response model (MGRM-C) was performed to estimate the item parameters, which was detailed as the equation below:

$$P_{ijk}=\frac{\exp\left(a_{i}\theta_{j}+d_{ik}\right)}{1+\exp\left(a_{i}\theta_{j}+d_{ik}\right)}$$

MGRM-C is a logistic probability model (*P*_*ijk*_) that examinee (*j*) will respond with category *k* (and above) of item *i* as a function of the item-category threshold (or easiness parameter, *d*_*ik*_), item discrimination parameter vector (*a*_*i*_), and examinee ability parameter vector (*θ*_*j*_). Log-likelihood, Akaike Information Criterion, Bayesian Information Criterion, and Sample-adjusted Bayesian Information Criterion were compared between Confirmatory Factor Analysis–based and Bifactor-based MIRT models. When the optimal model was chosen, multidimensional discrimination (< 0.5 indicates poor; 0.5–1.0 indicates moderate; 1.0–1.5 indicates good; > 1.5 indicates excellent) and multidimensional difficulty (monotonous distribution indicates good fitting) were used as the fitting indicators. In addition, item trace and item information surface were also visualized. Fourth, an iterative hybrid ordinal logistic approach with Monte Carlo Simulation was used to evaluate DIF between Americans and Chinese.^28^ *U*_*i*_ is defined as a discrete random variable representing the ordered item response to item *i*, whereas *u*_*i*_ (=0, 1, …, *m*_*i*_ − 1) is defined as the actual response to item *i*, with *m*_*i*_ ordered response categories. Based on the parallel regression assumption, regression coefficients are estimated for all cumulative logits with varying intercepts (*α*_*k*_). For each item, an intercept-only (null) model and three nested models are formed as follows:

$$Model\;0\;:logit\;P\left(u_{i}\geq k\right)=\alpha_{k}$$

$$Model\;1\;:logit\;P\left(u_{i}\geq k\right)=\alpha_{k}+\beta_{1}*ability$$

$${Model\;2\;:logit\;P\left(u_{i}\geq k\right)=\alpha_{k}+\beta }_{1}*ability+\beta_{2}*group$$

$${{Model\;3\;:logit\;P\left(u_{i}\geq k\right)=\alpha_{k}+\beta }_{1}*ability+\beta_{2}*group+\beta }_{2}*ability*group$$

where *P* (*u*_*i*_ ​≥ ​*k*) is defined as the cumulative probabilities that the actual item response *u*_*i*_ falls in category *k* or higher. Ability represents the latent trait measured by the test. Uniform DIF is evaluated by comparing the log likelihood values for Models 1 and 2, whereas nonuniform DIF is evaluated by comparing the log likelihood values for Models 2 and 3. An overall test of total DIF is estimated by comparing Models 1 and 3. Bonferroni corrected *P* values were set at 0.01/4 ​= ​0.0025 for the domain of Generic Elements and 0.01/6 ​= ​0.0017 for the domain of Shift-Persist. Fifth, two models, including Model 1: total score vs. Model 2: MIRT-based score, were compared based on AUC (Area Under Curve), NRI, IDI, calibration curves (estimated by Brier score), clinical impact curve, and decision curve analysis.^29^, ^30^, ^31^ NRI focuses on reclassification tables constructed separately for participants with and without events and quantifies the correct movement in categories, whereas IDI focuses on differences between sensitivities and “one minus specificities” for models with and without the new marker. All statistical analyses were performed by IRTPRO software and “lordif,” “mirt,” “riskRegression,” “nricens,” “PredictABEL,” “rms,” and “rmda” packages in R software.

## Results

### Demographics in the unmatched and matched data

**Fig. 1:**
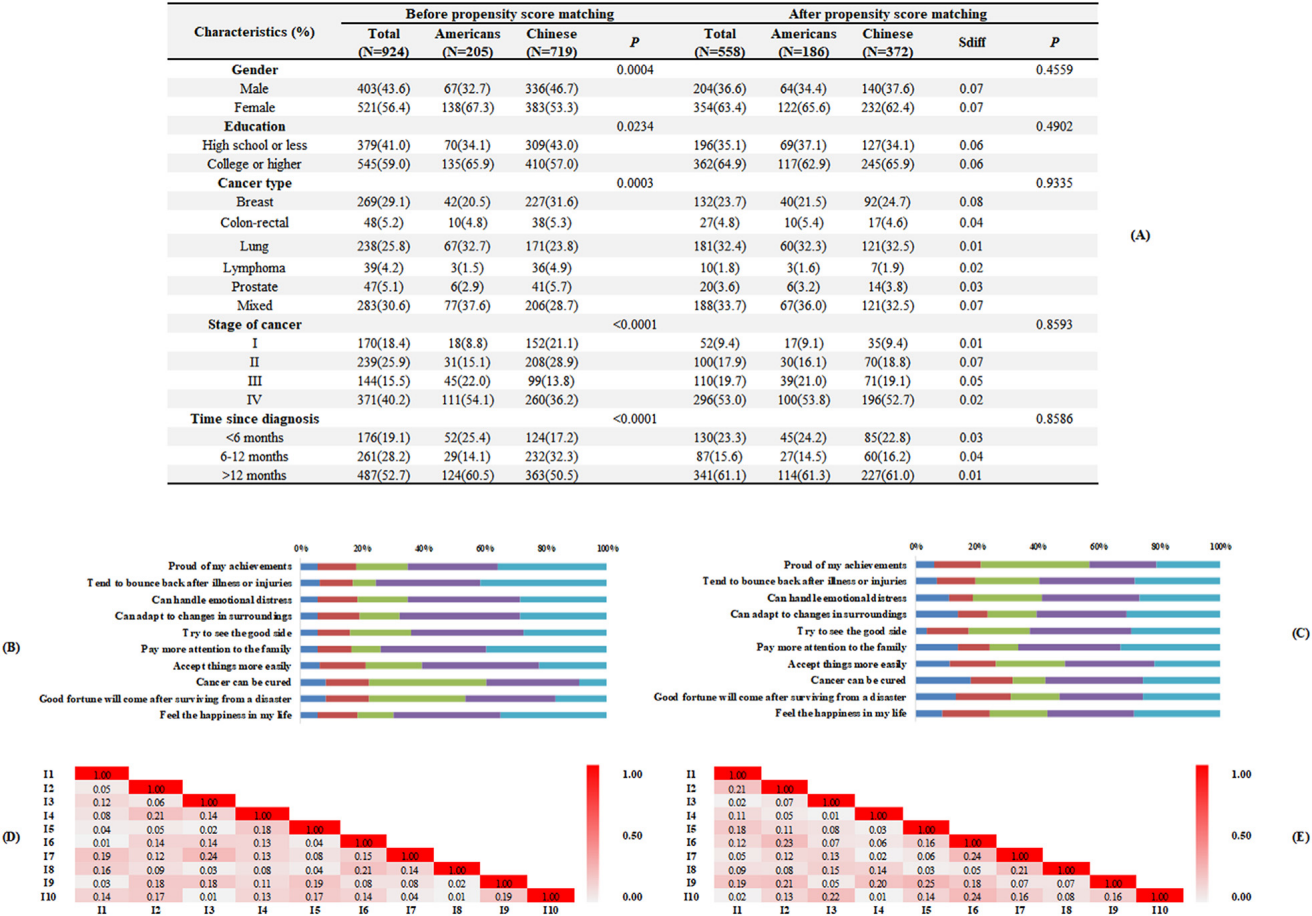
Demographics, item distribution, and local independence.

In the unmatched data set (*N* ​= ​924), mixed, breast, and lung cancer diagnoses accounted for 30.6%, 29.1%, and 25.8%, respectively. In Fig. 1A, before PSM, significant demographic differences were noted between Americans and Chinese in gender (*P* ​= ​0.0004), education (*P* ​= ​0.0234), cancer type (*P* ​= ​0.0003), stage of cancer (*P* ​< ​0.0001), and time since diagnosis (*P* ​< ​0.0001). After PSM, 186 Americans (90.7%) and 372 Chinese (51.7%) were included for further MIRT analysis, and the demographics were well balanced by small standardized differences (ranged from 0.01 to 0.08), which was presented in Fig. 1A.

### Item distribution and local independence

The item distribution for Americans and Chinese were summarized in Fig. 1B and C, respectively. In addition, item-pair local independence was visualized in Fig. 1D and E, indicating local independence was satisfied.

### Confirmatory factor analysis–based vs. bifactor-based MIRT models

**Fig. 2:**
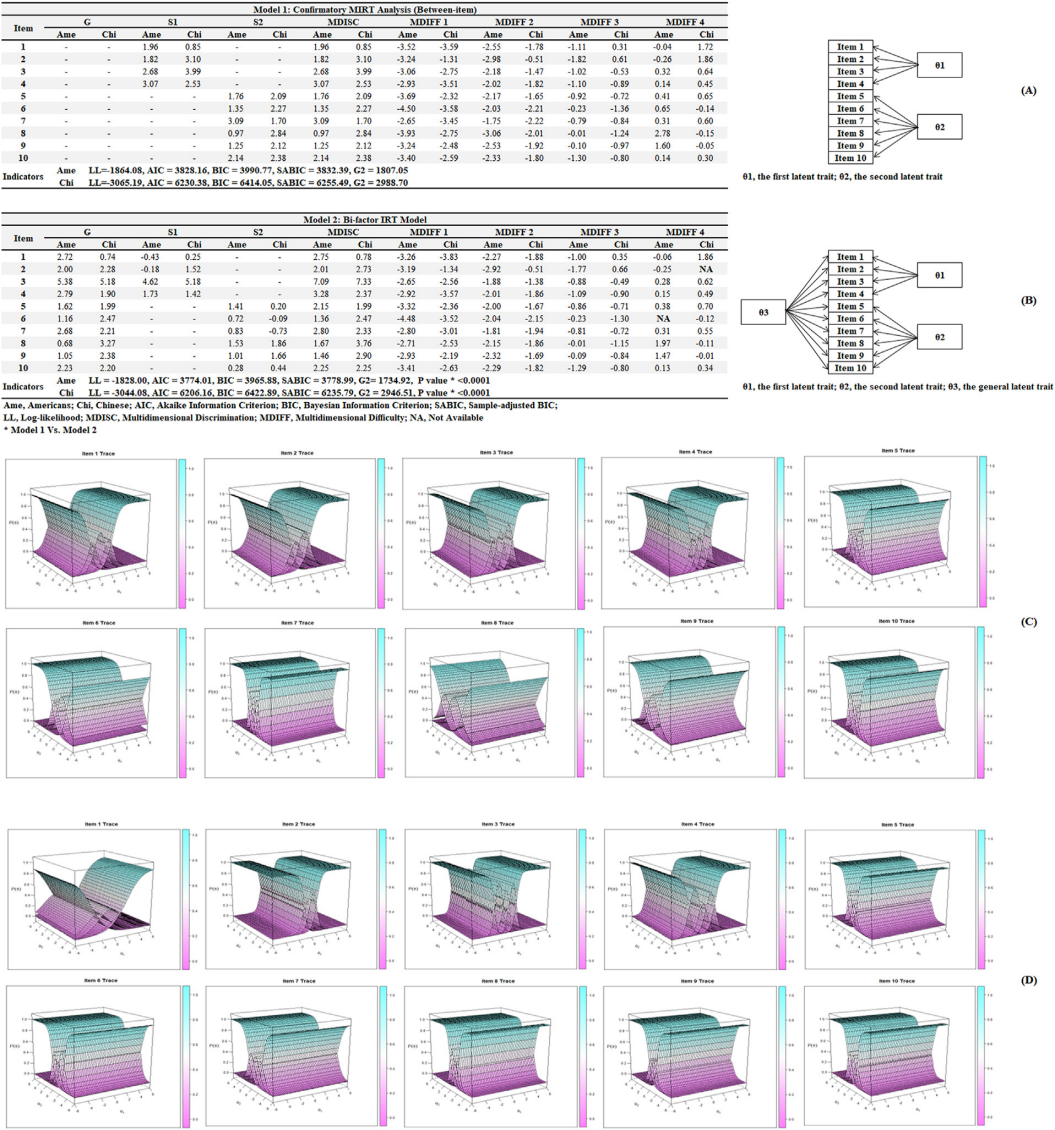
Confirmatory factor analysis–based vs. bifactor-based MIRT models.

**Fig. 3:**
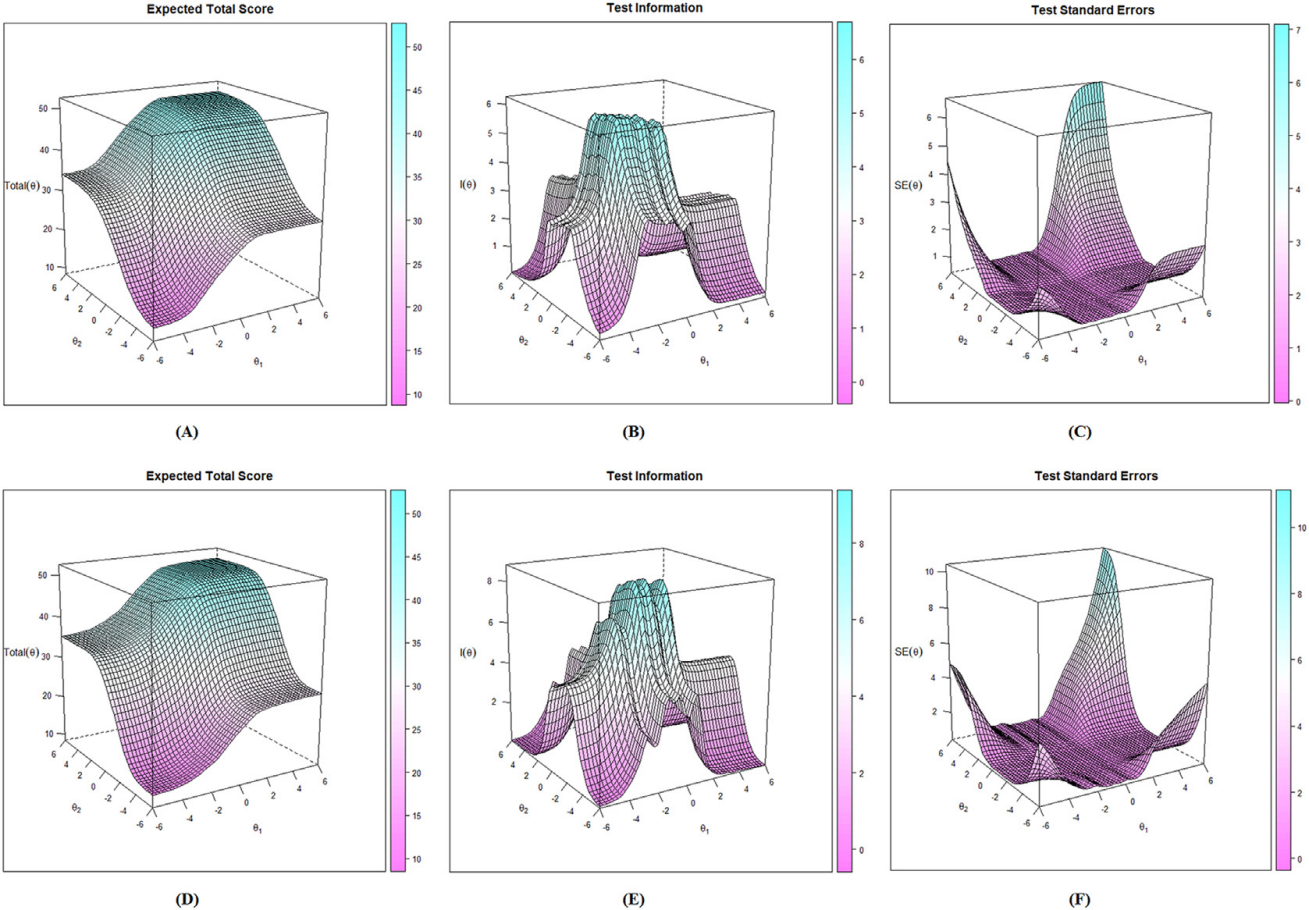
Expected total score, test information, and test standard errors.

Although better fitting indicators (*P* ​< ​0.0001 for both Americans and Chinese) were identified in the Bifactor-based MIRT model (Model 2, Fig. 2B) compared with the confirmatory factor analysis–based model (Model 1, Fig. 2A), negative slope values (S1 and S2) were recognized in Model 2 (ie, S1: −0.43 and −0.18 for items 1 and 2 in Americans, respectively; S2: −0.09 and −0.73 for items 6 and 7 in Chinese, respectively). In addition, disordered thresholds were identified in Americans (item 6, MDIFF4) and Chinese (item 2, MDIFF4), indicating the information overextraction. Thus, Confirmatory Factor Analysis–based MIRT model (Model 1) was chosen as the optimal model according to the parsimonious model guideline. In Fig. 2C and D, 10 item traces were visualized for Americans and Chinese, respectively, and monotonous distribution of theta values was confirmed. Finally, expected total score, test information, and test standard errors were summarized for Americans and Chinese in Fig. 3 and Fig. 3D–F, respectively.

### Differential item function

**Fig. 4:**
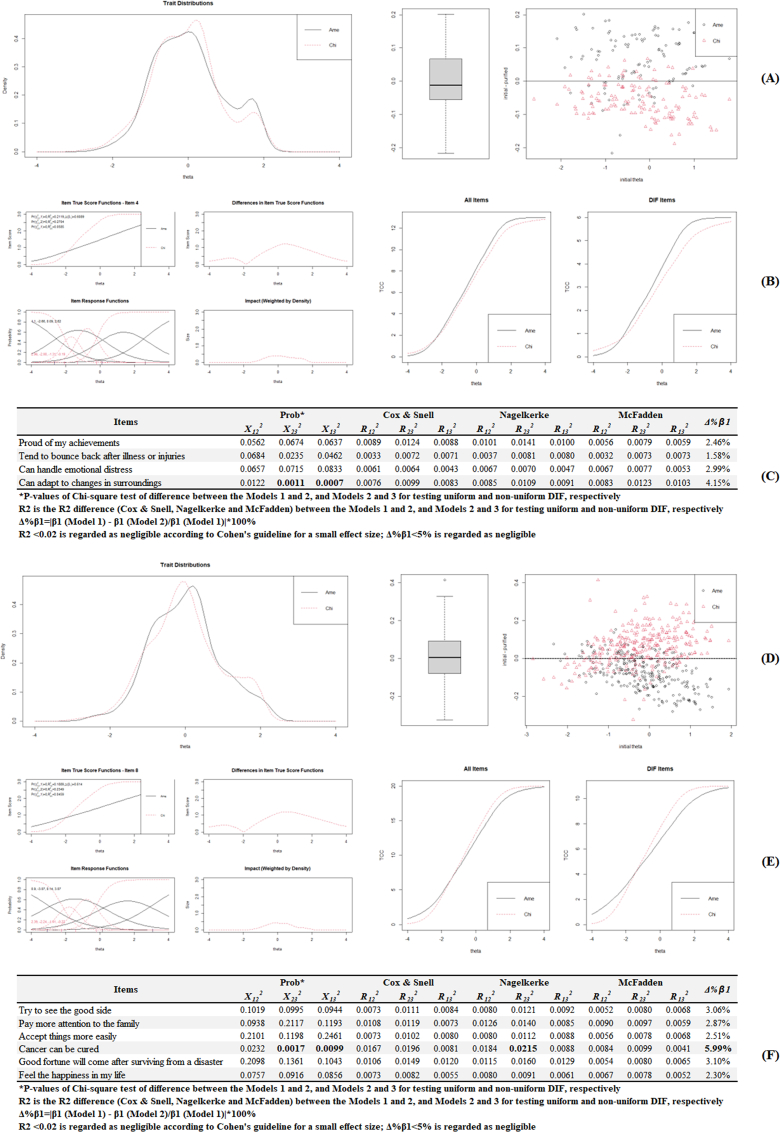
Differential item functioning using iterative hybrid ordinal logistic regression/item response theory and Monte–Carlo simulations.

For the domain of Generic Elements, Americans showed higher resilience compared with Chinese, although the difference was not statistically significant (Fig. 4A). At the individual level (Fig. 4B), all items except for item 4 showed no DIFs. According to Monte Carlo simulations–based thresholds in Fig. 4C, only nonuniform DIF was recognized in Item 4 (*P* ​= ​0.0011, Δ%β1 ​= ​4.15%). As for the domain of Shift-Persist, Americans showed lower resilience compared with Chinese, although the difference was not statistically significant (Fig. 4D). At the individual level (Fig. 4E), all items except for Item 8 showed no DIFs. According to Monte Carlo simulations–based thresholds in Fig. 4F, only nonuniform DIF was recognized in Item 8 (*P* ​= ​0.0017, Δ%β1 ​= ​5.99%).

### The comparison of different prediction models (total score vs. MIRT-based score)

**Fig. 5:**
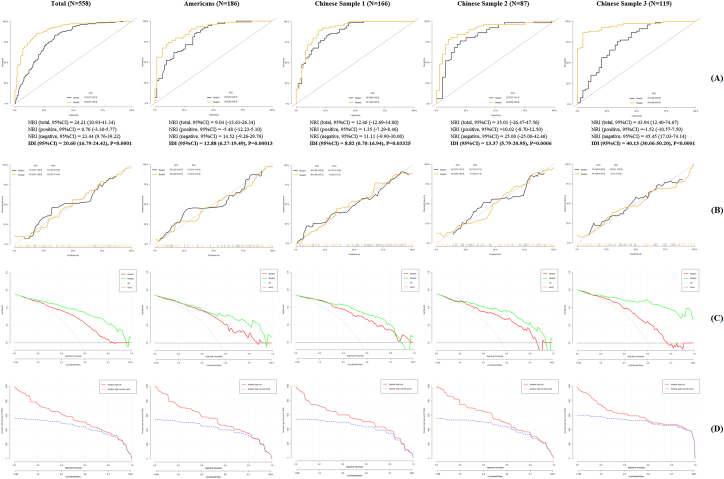
The comparison of different prediction models (total score vs. MIRT-based score).

Two models were developed to construct the prediction model for Distressed outcome, including Model 1: total score and Model 2: MIRT-based score. Compared with Model 1, AUC in Model 2 increased from 75.0%–86.5 to 89.2%–90.1% in four samples (Fig. 5A). NRI ranged from 9.04% to 35.01%, and IDI ranged from 8.82% to 20.60% (Fig. 5A). In Fig. 5B, brier scores in Model 2 ranged from 9.3 to 13.4, which were significantly less than those in Model 1 (ranged from 14.5 to 18.8). In Fig. 5C, DCA (Decision Curve Analysis) indicated that Model 2 showed higher net benefits compared with Model 1. Thus, Model 2 had better predictable ability to Distressed outcome than Model 1, and CICs about Model 2 clinical utilization in different samples were detailed in Fig. 5D.

## Discussion

In the present study, an ethnically diverse sample of Americans and Chinese was used to evaluate MI by comparing two languages: English and Mandarin. Before MI estimation, PSM was performed to help control the effect of potential confounders, resulting in a balanced demographic comparison, which was not well addressed in the previous research.^32^ The Confirmatory Factor Analysis–based MIRT model (between-item multidimensional theory framework) was better than the bifactor-based MIRT model (within-item multidimensional theory framework) in consideration of fitting indicators and information overextraction. It indicated that the underlying resilience constructs were conceptualized comparable in both the Americans and Chinese, and RS-SC-10 retained 10 items with monotonous thresholds and its original two-factor structure. In addition, a 5-point Likert scaling was suitable for RS-SC-10 in consideration of nondisordered thresholds identified in the present study. Although the local independence assumption was partly compromised owing to several high (> 0.2) item-pair residuals associations (ie, items 2 and 6, items 5 and 9, etc), the problematic item-pair proportions were small and could be ignored. In addition, test information revealed that RS-SC-10 could effectively distinguish patients with lower-middle or upper-middle resilience, confirming Hypothesis 1.

As for Hypothesis 2, nonuniform DIF were identified in Items 4 and 8, indicating that item-means equivalence was partially compromised in the level of resilience construct. For example, nonuniform DIF was recognized in Item 4, and we concluded that Americans had higher responses than Chinese when the patients had low resilience (from −4 to −2), whereas Chinese had higher responses than Americans when the patients had moderate-high resilience (from −2 to 4). Similar results could be derived from Item 8. Based on these findings, items 4 and 8 might not be suitable outcomes for cross-culturally resilience-related intervention between Americans and Chinese because only invariant items could be administered to compare language groups.^33^ In addition, more item parameters were estimated in a MIRT model than a unidimensional IRT model such as Rasch model, and a larger sample size should be warranted in the future research to replicate these findings especially for Americans in consideration of only 186 patients in the present study.^34^

As for Hypothesis 3, MIRT-based score vs. total score were compared and anchored against Distressed outcome based on a cross-sectional design. Similar NRI and IDI were identified between Americans and Chinese, and MIRT-based score had higher net benefits and better predictable abilities to Distressed outcome than resilience total score. Thus, MI could be indirectly confirmed by the Resilience-Distressed prediction model, and MIRT-based score instead of total score should be used in clinical settings to have a more precise resilience screening as well as a potential tool for predicting emotional distress. However, PRO-related anchors had subjective bias and external validations, for instance, diagnoses elicited from trained physician, objective indicators of cortisol, C-reactive protein, systolic pressure, and so on could be incorporated into MIRT validation research, which would provide more confidence to the interpretation of results.^35^^,^^36^

### Limitations

Several limitations should be considered. First, although PSM was performed to help control the effect of potential confounders, the sample size especially for Americans is inevitably reduced, resulting in a decreased statistical power in MIRT analysis.^37^^,^^38^ Therefore, the findings in the present study should be replicated in future research, especially with a larger sample of Americans. Second, the sample is not balanced based on the cancer stage, and more than half (53%) are patients with advanced-stage cancer (IV). Thus, these findings derived from the present study might not be generalized to patients with early cancer stage and should be further validated. Third, compensatory logistic multidimensional grade response model (MGRM-C) was performed in this study, indicating that a higher response in the domain of Generic could compensate a lower response in the domain of Shift-Persist (linear accumulation), and this model might not be consistent with resilience construct resulting in biased parameter evaluations.^39^ A noncompensatory MGRM, which means the probability is the product of probabilities derived from Generic and Shift-Persist, is recommended to test the robustness of findings in future research.^39^ Fourth, in the present study, MI was estimated by IRT-based methods, and these findings should be further confirmed and compared by Classic Test Theory–based factor model in the future research.^40^

## Conclusions

Although partial MI has been identified between Americans and Chinese, RS-SC-10 remains a critical indicator to emotional distress in cancer care.

## Data Availability

The data that support the findings of this study are available on request from the corresponding author. The data are not publicly available due to privacy or ethical restrictions.
