# Supplementary material for: Measurement invariance of the 10-item resilience scale specific to cancer in Americans and Chinese: A propensity score–based multidimensional item response theory analysis
Source: Asia Pac J Oncol Nurs. 2022 Nov 26;10(2):100171. doi: 10.1016/j.apjon.2022.100171 (PMC9827362; doi:10.1016/j.apjon.2022.100171)
Supplement: Multimedia component 1 [file mmc1.docx]

**Table S1. 10-item Resilience Scale Specific for Cancer (RS-SC-10)**

**Instructions:** Please read the items below and indicate how often you agree with these statements over the last 4 weeks. If some specific situations did not occur, imagine about how you would feel if it had happened. There are no right or wrong answers and please circle on the number which most closely described your feelings.

| **Item** | **Content** | **Never** | **Seldom** | **Sometimes** | **Often** | **Always** |
| --- | --- | --- | --- | --- | --- | --- |
| 1 | Proud of my achievements | 1 | 2 | 3 | 4 | 5 |
| 2 | Tend to bounce back after illness or injuries | 1 | 2 | 3 | 4 | 5 |
| 3 | Can handle emotional distress | 1 | 2 | 3 | 4 | 5 |
| 4 | Can adapt to changes in my surroundings | 1 | 2 | 3 | 4 | 5 |
|  | **When you are faced by cancer,** |  |  |  |  |  |
| 5 | Try to see the good side | 1 | 2 | 3 | 4 | 5 |
| 6 | Pay more attention to family | 1 | 2 | 3 | 4 | 5 |
| 7 | Accept things more easily | 1 | 2 | 3 | 4 | 5 |
| 8 | Cancer can be cured | 1 | 2 | 3 | 4 | 5 |
| 9 | I believe that good fortune will come after surviving a disaster | 1 | 2 | 3 | 4 | 5 |
| 10 | Feel the happiness in my life | 1 | 2 | 3 | 4 | 5 |
